# A Moribund Community: Are the Citizens of Worcester Unique?

**Published:** 1913-08-30

**Authors:** 


### A MORIBUND COMMUNITY.

### Are the Citizens of Worcester Unique?

This question arises out of an article which appears in the *Worcester Times* of the 23rd inst., based upon our comments on the condition of hospital matters at Worcester. We ventured to ask last week if the Mayor or the Bishop or the Lord Lieutenant of the county would put his hand to the plough and give the citizens a real, up-to-date, modern hospital? This question has caused the *Worcester Times* some searchings of heart, for it insists that the three dignitaries in question have each and all by earnest endeavour striven and failed "to rouse Worcester and the Worcestershire people to a more adequate recognition of the claims and needs of the infirmary." This argument, if pushed home, must mean in fact that the citizens of Worcester and the residents in the county of Worcester do not adequately support, as all other citizens and residents do in other counties and cities throughout the country, the hospital which ministers to the needs of the whole community. It would have been cruel, if not improper, for a non-resident to have expressed a fear even that such a universal indifference and parsimony prevailed at Worcester, but as the local *Times* infers this to be the case, the hospital world must with amazement accept it as true.

When neither the Bishop nor the Lord Lieutenant nor the Mayor can exert any influence whatever of a practical kind to help the sick in their own particular county and city there must be something radically wrong somewhere. What is at the bottom of this evil? Is it parsimony, the want of spiritual apprehension, the fact that the parable of the Good Samaritan falls upon deaf ears, or is it that the citizens feel that the administration of their local infirmary is so feeble and has been so lamentable in its results for many years that on the whole the best way to mend this state of affairs is to end the infirmary and close its doors?

As the *Worcester Times* has been courageous enough to condemn by implication the citizens and the county residents of Worcester, perhaps its editor will do a further public service by stating his own opinion quite frankly as to which of the grounds enumerated is in fact the cause of the unfortunate and unique neglect of the claims of the sick at Worcester to-day. We can at any rate assure him that the reasons he has given for the impotence of the dignitaries we mentioned last week, if, as we are bound to believe they are, sound and true, must direct the attention of British people to this moribund community in Worcester, for it is without a parallel in the British Islands, or, as far as our observation goes, in any other Christian community throughout the world. It is fair to assume that the prosperity and means of the average citizens and inhabitants of Worcester are, in fact, at least equal to those of the residents in other similar cities and counties, where the citizens take a pride in contributing liberally and adequately to the support of their local medical charities.

Why then do they remain indifferent and asleep in hospital matters? It has been suggested that possibly the citizens of Worcester are mostly Christian Scientists and do not in consequence believe in medical treatment or hospital care of any kind for the sick. Another suggestion is that the air and hygienic condition of the city of Worcester are so magnificent that the citizens regard themselves as above the necessity of putting their hands in their pockets to provide for the adequate administration and upkeep of the Worcester Infirmary. The latter suggestion does not commend itself to our judgment, seeing that the last time we visited Worcester we were certainly not specially impressed by its sanitary administration or hygienic condition. The present is the holiday season, and a humourist who is enjoying the sunshine and the sea far from the madding crowd holds to the opinion that Worcester is one of those places where the majority have no business of any kind. Hence the citizens have become so absorbed in holiday-making that they have no time for the adequate discharge of the responsible duties of daily life. Seriously we put this question to the *Worcester Times* and, through the local Press, to the citizens of Worcester: How can you good people of Worcester city and county consent to remain inactive whilst your hospital is being more fatally weakened in the financial sense every year for want of a little resolute help and self-denial on your part whose privilege it should be gladly to unite and make the Worcester Infirmary once and for all up to date and worthy of the best traditions of your ancient city?

